# Lactate promotes myogenesis via activating H3K9 lactylation‐dependent up‐regulation of Neu2 expression

**DOI:** 10.1002/jcsm.13363

**Published:** 2023-11-02

**Authors:** Weilong Dai, Gang Wu, Ke Liu, Qianqian Chen, Jingli Tao, Honglin Liu, Ming Shen

**Affiliations:** ^1^ Department of Animal Genetics, Breeding and Reproduction, College of Animal Science and Technology Nanjing Agricultural University Nanjing China

**Keywords:** Histone lactylation, Lactate, Myoblast differentiation

## Abstract

**Background:**

Lactate, a glycolytic metabolite mainly produced in muscles, has been suggested to regulate myoblast differentiation, although the underlying mechanism remains elusive. Recently, lactate‐mediated histone lactylation is identified as a novel epigenetic modification that promotes gene transcription.

**Methods:**

We used mouse C2C12 cell line and 2‐month‐old male mice as in vitro and in vivo models, respectively. These models were treated with lactate to explore the biological function and latent mechanism of lactate‐derived histone lactylation on myogenic differentiation by quantitative real‐time PCR, western blotting, immunofluorescence staining, chromatin immunoprecipitation, cleavage under targets and tagmentation assay and RNA sequencing.

**Results:**

Using immunofluorescence staining and western blotting, we proposed that lactylation might occur in the histones. Inhibition of lactate production or intake both impaired myoblast differentiation, accompanied by diminished lactylation in the histones. Using lactylation site‐specific antibodies, we demonstrated that lactate preferentially increased H3K9 lactylation (H3K9la) during myoblast differentiation (CT VS 5, 10, 15, 20, 25 mM lactate treatment, *P* = 0.0012, *P =* 0.0007, and the rest of all *P* < 0.0001). Notably, inhibiting H3K9la using P300 antagonist could block lactate‐induced myogenesis. Through combined omics analysis using cleavage under targets and tagmentation assay and RNA sequencing, we further identified *Neu2* as a potential target gene of H3K9la. IGV software analysis (*P* = 0.0013) and chromatin immunoprecipitation‐qPCR assay (H3K9la %Input, LA group = 9.0076, control group = 2.7184, IgG = 0.3209) confirmed that H3K9la is enriched in the promoter region of *Neu2*. Moreover, siRNAs or inhibitors against Neu2 both abrogated myoblast differentiation despite lactate treatment, suggesting that Neu2 is required for lactate‐mediated myoblast differentiation.

**Conclusions:**

Our findings provide novel understanding of histone lysine lactylation, suggesting its role in myogenesis, and as potential therapeutic targets for muscle diseases.

## Introduction

Skeletal muscle is the largest tissue in the healthy human body, constituting 40% of the whole‐body weight.^1^ The number of myofibres in mammals are determined at the embryonic stage, while postnatal muscle growth only involves hypertrophy of myofibres, rather than numerical growth.^2^ When skeletal muscle is injured, muscle stem cells, which are distributed on the basement membrane and plasma membrane of the myofibre, will be activated, switching from quiescence to proliferation and differentiation, thereby activating muscle regeneration.^3^ Recent studies suggested that lactate could promote myoblast differentiation in vitro and muscle regeneration in vivo.^4^, ^5^, ^6^, ^7^ However, few studies have examined the mechanism of lactate‐induced myogenesis.

Lactate has long been considered as a metabolic byproduct. Increasing evidence, however, indicates that lactate is a multifunctional molecule involved in the regulation of a wide spectrum of biological processes including mitochondrial respiration, gluconeogenesis, and signalling transduction.^8^ Recent findings by Zhang et al. reported that lactate contributed to epigenetic regulation of genes expression by lactylation lysine residues of histone. This study suggested that histone H3 lysine lactylation (Kla) mediates polarization of macrophages derived from mouse bone marrow, and the lactylation level is paralleled to the intracellular lactate levels.^9^ Of note, the choice of cell fate by muscle stem cells for self‐renew or differentiate into the muscle lineage is governed by posttranslational modifications of histones.^10^ However, it is unknown whether lysine lactylation occurs during myogenesis.

Myogenesis in vivo and in vitro both require the withdrawal of myoblast from cell cycle and terminal differentiation into myotubes. Evidence suggested that glucose metabolites are required for muscle regeneration.^11^ However, muscle stem cells or myoblasts are not very efficient to use glucose due to their dominant glycolytic metabolism. The process of myoblast differentiation is associated with metabolic reprogramming, as reflected by improved efficiency of glucose metabolism in myotube.^12^ Upon transition of myoblasts into myotubes, the altered metabolic patterns might cause changes in lactate levels.^11^ Of note, various histone acylation derived from glucose metabolites (such as histone acetylation) have been shown to play a key role in myogenic differentiation.^13^ Inspired by recent discoveries of histone lactylation modification,^14^ we asked whether lactate might affect myoblast differentiation through histone lactylation‐mediated gene expression.

Sialylation is a post‐translational modification regulated by sialyltransferases and sialidases in the terminal residues of many glycoprotein complexes.^15^ Four types of mammalian sialidases have been identified and designated as neuraminidase 1 (Neu1), neuraminidase 2 (Neu2), neuraminidase 3 (Neu3) and neuraminidase 4 (Neu4). They are encoded by different genes and differ in major subcellular localization and enzymatic properties.^16^ Neu2 is mainly distributed in the cytoplasm and functions specifically to hydrolyse sialic acid residues in glycoconjugate.^15^ Neu2 was recognized as a myogenesis positive factor on skeletal muscle.^17^, ^18^, ^19^ During myoblast differentiation, both mRNA and protein expression of Neu2 is gradually increased.^17^ Previous studies have shown that overexpression of Neu2 promoted myoblast differentiation.^17^ Knocking out Neu2 impaired muscle function and caused abnormal lipid metabolism in mouse.^18^ The transcription starts site (TSS) of the *Neu2* gene contains two E‐box sequences, which is the binding site for the family of myogenic regulatory factors (MRFs).^19^ Therefore, MRFs‐induced myoblast differentiation is in part through activating *Neu2* expression.

In this study, we first identified the correlation between histone lysine lactylation and myoblast differentiation. Elevated lactate levels efficiently promoted histone lactylation, induced myoblast differentiation in vitro and facilitated muscle regeneration in vivo. By contrast, inhibition of histone lactylation suppressed myotube formation. Mechanistically, lactate preferentially increased H3K9 lactylation, leading to enhanced transcription of *Neu2*, which is suggested to stimulate myoblast differentiation. Taken together, our data revealed a novel mechanism involving histone lactylation in lactate‐mediated myogenesis.

## Materials and methods

### Reagent

AZD3965 (#S7339), R‐GNE‐140 (#S6675) and C646 (#S7152) were obtained from Selleck Chemicals LLC, Houston, TX, USA. Neu2 inhibiter DANA (#HY‐125798) was purchased from MedChemExpress. The remaining reagents were purchased from Sigma‐Aldrich.

### Cell culture and differentiation

Mouse myoblast C2C12 cells were purchased from The Chinese Academy of Sciences cell bank (Shanghai, China) and grown in incubators at 37°C, 5% CO_2_. Proliferating cells were cultured in high‐glucose Dulbecco's modified Eagle's medium added with 1% penicillin–streptomycin and 10% fetal bovine serum. After C2C12 cells grown to 80–90% confluence, cells were cultured in Dulbecco's modified Eagle's medium with 1% penicillin–streptomycin and 2% horse serum for myogenic differentiation.

### Cell treatment

C2C12 were treated with the addition of either 15 mM L‐lactate or sodium lactate with differentiation medium changes every 24 h for 3–5 days. To inhibit activity of lactate dehydrogenase A (LDHA), C2C12 cells were cultured with (R)‐GNE‐140 (10 μM) or dimethyl sulfoxide for 3 days. To block lactate uptake, myoblasts were treated with AZD3965 (0, 25, 50 and 100 nM) for 3 days. In order to inhibit function of Neu2, C2C12 cells were treated with DANA (50 μM) and cultured in differentiation medium for 3 days.

### Western blotting

Proteins were extracted from cells and muscle tissue by radioimmunoprecipitation assay buffer with 1% phenylmethylsulfonyl fluoride. Western blotting was performed according to previously reported method.^20^ The antibody products and dilution conditions used in this experiment were showed in Table S3.

### Immunofluorescence staining

The method as previously described.^20^ The antibodies used were listed in Table S3.

### Transfection of small interfering RNA (siRNA)

LDHA siRNAs and negative control were obtained from GenePharma Inc (Shanghai, China). The sequences of LDHA siRNAs were as follows (5′‐3′): si‐1: GCCAUCAGUAUCUUAAUGATT; si‐2: GUCUCCCUGAAGUCUCUUATT; si‐3 GGGUCUCUAUGGAAUCAAUTT. Transient transfections of siRNAs were conducted with Lipofectamine 3000 (Invitrogen, USA) following manufacturer conditions. After 6–12 h, replaced medium with 2% horse serum to differentiation for 3–4 days.

### Cell viability assay

The myoblasts were evenly plated in 96 well plates first; after cell density grown to 50%, the different concentrations of lactate were added for 12 or 24 h. Then we replaced medium and cultured with 100 μL Cell Counting Kit‐8 (CCK‐8) in the incubator for 2 h.

### RNA isolation and quantitative real‐time PCR (qRT‐PCR)

The method as previously described.^20^ Primers used for qRT‐PCR were showed in Table S1.

### RNA sequencing (RNA‐seq)

Total RNA was extracted from C2C12 cells. A total amount of 2 μg RNA per sample was used as input material for the RNA sample preparations. Sequencing libraries were generated using NEBNext® Ultra™ RNA Library Prep Kit for Illumina® (#E7530L, NEB, USA) following the manufacturer's recommendations and index codes were added to attribute sequences to each sample. Briefly, mRNA was purified from total RNA using poly‐T oligo‐attached magnetic beads. Fragmentation was carried out using divalent cations under elevated temperature in NEBNext First Strand Synthesis Reaction Buffer (5×). First strand cDNA was synthesized using random hexamer primer and RNase H. Second strand cDNA synthesis was subsequently performed using buffer, dNTPs, DNA polymerase I and RNase H. The library fragments were purified with QiaQuick PCR kits and elution with EB buffer, and then terminal repair, A‐tailing and adapter added were implemented. The aimed products were retrieved, and PCR was performed, and then the library was completed. The libraries were sequenced by Annoroad using the Illumina platform and 150 bp paired‐end reads were generated.

### Cleavage under targets and tagmentation (CUT&Tag) assay

The CUT&Tag assay was performed using the NovoNGS CUT&Tag 2.0 High‐Sensitivity Kit (NovoProtein, N259‐YH01); 1 × 10^5^ C2C12 cells were washed twice with 1.5 mL of wash buffer and then mixed with activated concanavalin A beads. After incubations with the primary antibody (H3K9la, room temperature, 2 h) and secondary antibody (room temperature, 1.5 h), the cells were washed and incubated with pAG‐Tn5 for 1 h. Then, tagmentation buffer was added to activate tagmentation for 1 h. Next, we added a stop buffer at 55°C for 10 min to stop tagmentation. DNA fragments were extracted by Tagment DNA extract beads. The libraries were sequenced by Annoroad using the Illumina Novaseq platform.

### Chromatin immunoprecipitation

Chromatin immunoprecipitation (ChIP) was prepared as previously described.^21^, ^22^ ChIP‐qPCR primers were showed in Table S2.

### Mouse experiments

Two‐month‐old male ICR mice were obtained from Qinglongshan (China, Nanjing), and mice were allocated randomly to experimental groups and processed independent on size, body weight or age, and all procedures with animals were conducted in accordance with the guidelines of the Animal Research Institute Committee at Nanjing Agricultural University (SYXK‐2017‐0027). To induce muscle regeneration, 30 μL of 1.2% BaCl_2_ was intramuscularly injected into the tibialis anterior (TA) muscle. Then, mice were daily intraperitoneal injection with sodium lactate (500 mg/kg) or the equal volume of saline as the control group for 7 days. Regenerating TA muscles was harvested at day 1, 4 and 7 post‐injuries.

### Haematoxylin and eosin (H&E) staining

TA muscles were harvested and fixed in 4% paraformaldehyde for 72 h and subsequently embedded in paraffin. For the assessment of muscle morphology, 4 μm thick cross‐sections of TA muscles were subjected to H&E staining, which was performed according to procedures provided by the H&E staining kit.

### Statistical analysis

Statistical analysis was performed using Prism 8 software (GraphPad Software, San Diego, CA); Student's *t*‐test and one‐way ANOVA were used to analyse the statistical significance between two groups. Data are expressed as means ± SEM of three independent experiments. *P < 0.05, **P < 0.01, and ***P < 0.001; not significant (ns).

## Results

### Lactate promotes myoblast differentiation and histone lysine lactylation

**Figure 1:**
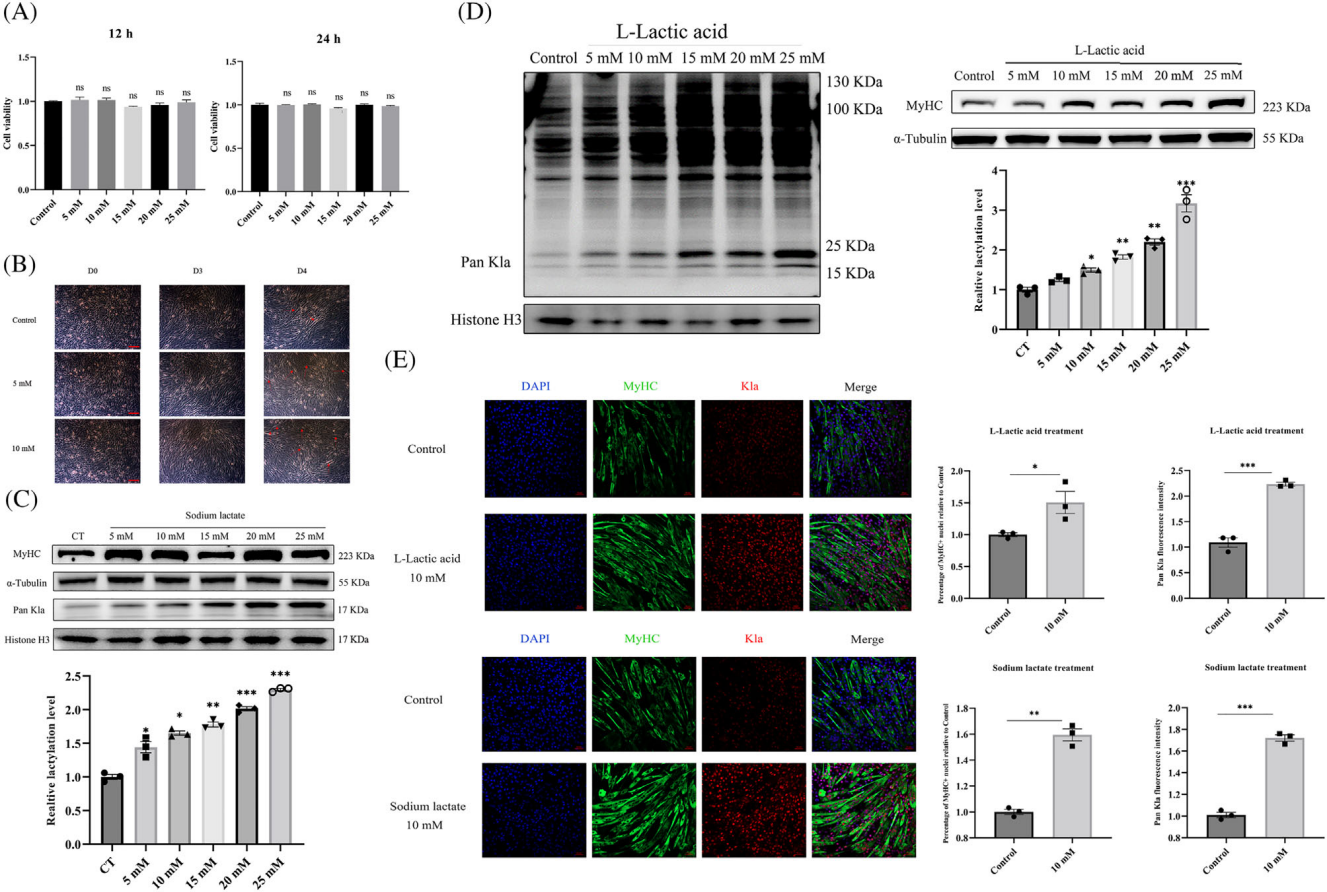
Lactate elevated histone lysine lactylation and enhance myoblast differentiation. (A) C2C12 cells were treated with (0, 5, 10, 15, 20 and 25 mM) and cultured with GM for 12 and 24 h, and cell viability was then determined using the CCK‐8. (B) Morphological photographs of C2C12 treated with 5 and 10 mM L‐lactate under a light microscope were taken of cells differentiated for 1, 3 and 4 days. Scale bars correspond to 100 μm. (C) Western blot of MyHC differentiated for 4 days with sodium lactate treatment. Protein quantification plots were analysed by ImageJ for grey scale value mapping. (D) Lactylation and MyHC protein levels were detected in differentiation concentration L‐lactate treated on C2C12 cells by western blot. Protein quantification plots were analysed by ImageJ for grey scale value mapping. (E) Representative photographs of MyHC, Kla immunofluorescence staining in C2C12 cells differentiated for 4 days under L‐lactate (10 mM) or sodium lactate (10 mM) treatment. Scale bar = 100 μm. And quantitation of the MyHC, Kla positive cells (MyHC+ is the proportion of MyHC positive cells with at least one nucleus). All data are expressed as mean values ± SEM; *n* = 3 biological replicates in each group. *P < 0.05, **P < 0.01, ***P < 0.001.

We initially examined the impact of lactate on myoblast proliferation by CCK‐8 assay. As shown in Figure 1A, no significant changes in cell proliferation rate were observed following lactate treatment. We also examined the effect of lactate on the morphological differentiation of myoblasts. As shown in Figure 1B, compared with control cells, lactate treatment cells indicate a better morphology of myoblast differentiation. In addition, the protein level of myosin heavy chain (MyHC, a myogenic differentiation marker) and the amount of myotube formation were significant elevated after L‐lactate/sodium lactate treatment (Figure 1C–E), consistent with previous findings.^6^ We next perform western blot to determine whether L‐lactate or sodium lactate treatment might affect protein lactylation. Using anti‐Pan Kla, we found that lactate significantly increased the level of lactylated proteins around 15–20 KDa (Figure 1D), an area corresponding to the molecular weight of H3 histone.^23^ Immunofluorescence staining results showed that lactate‐induced lactylation was predominantly distributed within the nucleus, further indicating that lactylation might occur in histone proteins (Figure 1E).

### Inhibition of lactate production prevents histone lactylation and muscle differentiation

**Figure 2:**
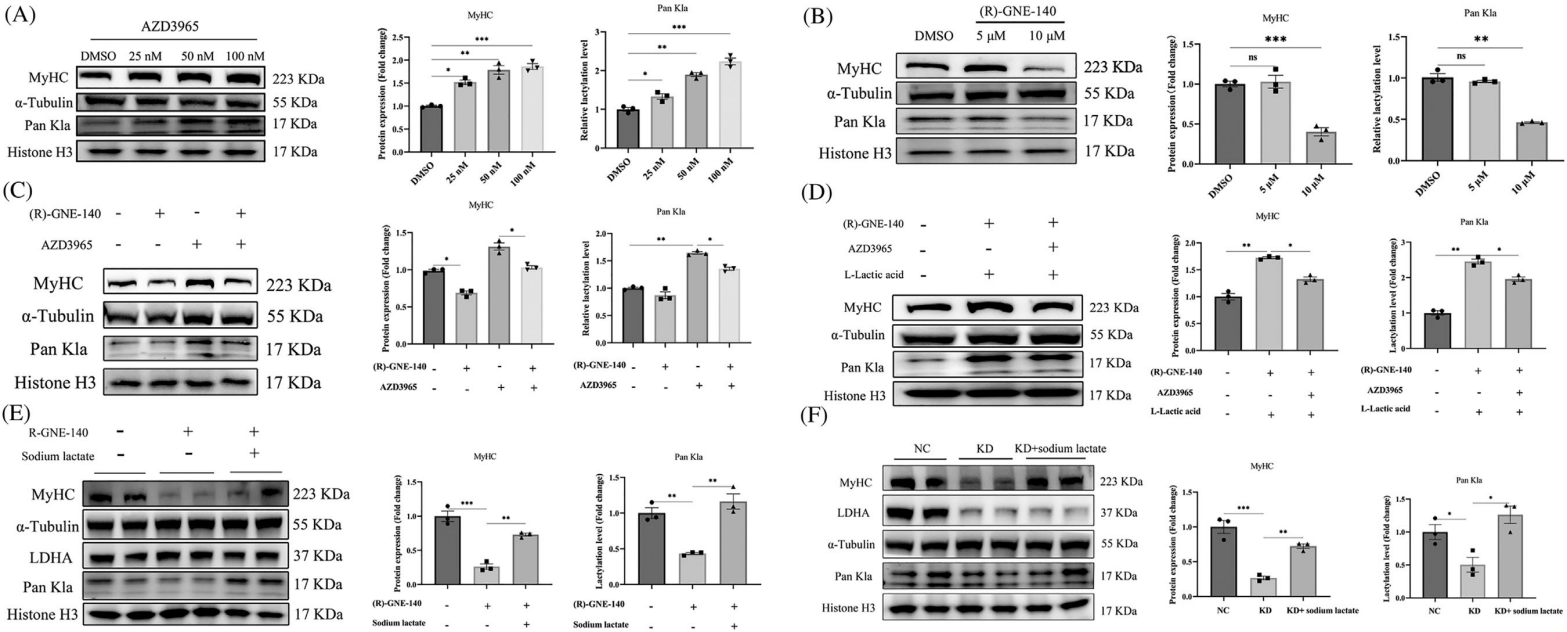
Altering intracellular lactate level in myoblasts regulate lactylation and differentiation. (A) C2C12 cells differentiation for 4 days upon AZD3965 treatment, then detected MyHC protein level and histone lactylation level by western blot. And quantitation of the MyHC, Kla protein expression level relative to α‐tubulin and histone H3. (B) C2C12 cells cultured with *(R)*‐GNE‐140 (5 and 10 μM) and differentiation for 4 days, then detected MyHC protein level and histone lactylation level by western blot. And quantitation of the MyHC, Kla protein expression level relative to α‐tubulin and histone H3. (C) (R)‐GNE‐140 (10 μM) and AZD3965 (100 nM) co‐treated on C2C12 cells and differentiation for 4 days, then detected MyHC protein expression level and histone lactylation level by western blot. And quantitation of the MyHC, Kla protein expression level relative to α‐tubulin and histone H3. (D) After inhibiting MCTs and LDHA, induced C2C12 cells differentiation for 4 days with lactate (15 mM) were added, then detected MyHC and histone lactylation protein expression level by western blot. And quantitation of the MyHC, Kla protein expression level relative to α‐tubulin and histone H3. (e) After inhibit LDHA function, then suppled with lactate (15 mM) to differentiation for 4 days, then detected MyHC protein level and histone lactylation by western blot. And quantitation of the MyHC, Kla protein expression level relative to α‐tubulin and histone H3. (f) After knock down LDHA expression, then added lactate (15 mM) to rescue C2C12 differentiation for 4 days. MyHC protein level and histone lactylation level detected by western blot. And quantitation of the LDHA, MyHC, Kla protein expression level relative to α‐tubulin and histone H3. All data are expressed as mean values ± SEM; *n* = 3 biological replicates in each group. *P < 0.05, **P < 0.01, ***P < 0.001.

Monocarboxylate transporters (MCTs) are responsible for the transport of monocarboxylic acids, such as lactate, across the cell membrane.^24^ Monocarboxylate transporters 1 (MCT1) primarily mediates lactate uptake on muscle cells for oxidative respiration, while monocarboxylate transporters 4 (MCT4) has a greater propensity for lactate export.^25^ To further elucidate the relationship among lactate, histone lactation and myoblast differentiation, we treated myoblast with the MCTs (MCT1 and MCT4) inhibitor AZD3965, which was reported to inhibit lactate export and up‐regulate lactate level in melanomas cells.^26^ As shown in Figure 2A, cells received AZD3965 treatment exhibited markedly elevated levels of MyHC protein and histone lysine lactylation. We then blocked lactate production using (R)‐GNE‐140, a specific antagonist of lactate dehydrogenase (LDH). Western blot assay showed that 10 μM (R)‐GNE‐140 treatment could significantly reduce both MyHC expression and histone lysine lactylation (Figure 2B). (R)‐GNE‐140 also blocked AZD3965‐induced accumulation of endogenous lactate, histone lysine lactylation and MyHC expression (Figure 2C). To further confirm whether lactylation is required for lactate‐induced myoblast differentiation, we inhibited exogenous lactate intake using MCTs antagonists. Our results showed that lactate‐stimulated up‐regulation of MyHC expression and histone lactylation was abrogated following inhibition of MCTs (Figure 2D). Furthermore, to rule out the nonspecific effects of (R)‐GNE‐140, we designed three siRNAs of lactate dehydrogenase A (LDHA) to repress endogenous lactate production during myoblast differentiation (Figure S1a,b), and we selected the siRNA#3 for our next experiments. Knocking down LDHA expression with siRNA#3 showed similar results with LDH inhibitor, as reflected by a remarkable reduction in differentiation activity and histone lactylation level (Figure S1c–f).

We next performed a rescue experiment to demonstrate whether lactate level is a direct reason affecting myoblast differentiation and histone lysine lactylation. After inhibiting LDH activity with RNA interference or (R)‐GNE‐140, myoblast cells were cultured with 15 mM sodium lactate for 4 days. Our results showed that supplementation with sodium lactate not only restored the differentiation activity of myoblast but also prevented the decline of MyHC protein expression and histone lysine lactylation in cells treated with siRNA or inhibitor (Figure 2E,F). These results suggested that lactate‐triggered histone lactylation might contribute to myoblast differentiation.

### Lactate‐induced H3K9 lactylation modification promotes myoblast differentiation

**Figure 3:**
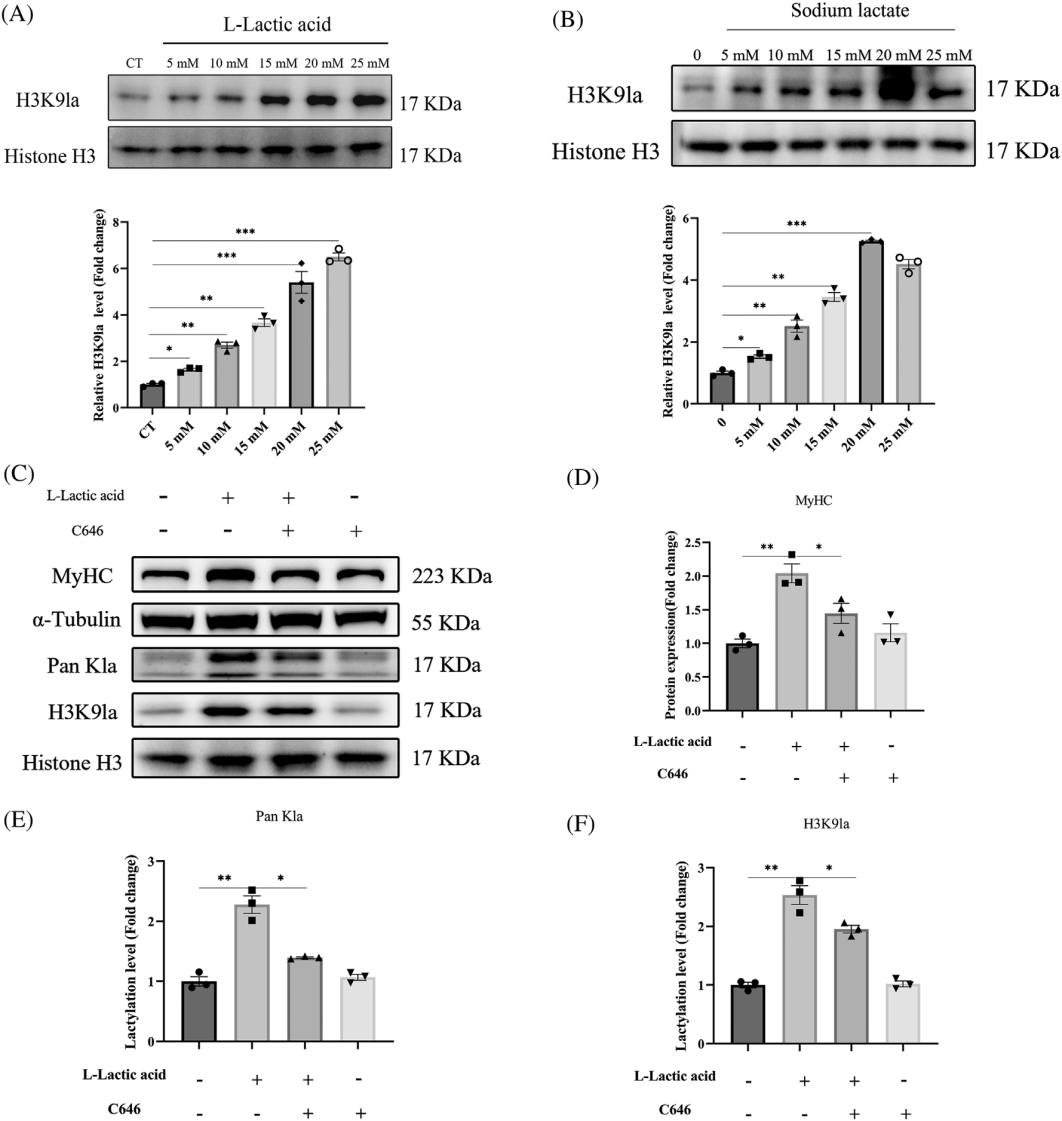
Lactate treatment promote H3K9la level. (A) C2C12 cells treated with L‐lactate and cultured with DM for differentiation 4 days, then detected H3K9la by specific antibody anti‐H3K9la by western blot. H3K9 lactylation level relative to histone H3. (B) C2C12 cells treated with sodium lactate and cultured with DM for differentiation 4 days, then deterimend H3K9la by specific antibody anti‐H3K9la by western blot. H3K9 lactylation level relative to histone H3. (C) C2C12 cells treated with C646 (10 μM), then cultured with L‐lactate (15 mM) for differentiation 4 days, then detected H3K9la by specific antibody anti‐H3K9la by western blot. (D–F) Quantitation of the MyHC, Kla, H3K9la protein expression level relative to α‐tubulin and histone H3 in the C. all data are expressed as mean values ± SEM; *n* = 3 biological replicates in each group. *P < 0.05, **P < 0.01, ***P < 0.001.

To further explore which type of histone lactylation might be involved in muscle differentiation, we conducted western blot assay using lactylation site‐specific antibodies like H3K14la, H3K9la and H3K18la. Remarkably, both L‐lactate and sodium lactate treatment could preferentially enhance H3K9 lactylation in a dose‐dependent manner (Figure 3A,B, Figure S2a,b), which is consistent with the result of Pan Kla as shown in Figure 1D. To further confirm the role of H3K9 lactylation in myoblast differentiation, we treated myoblast cells with C646, an inhibitor of P300, which was reported as a major lactylation writer.^27^, ^28^ The western blot results showed that the P300 inhibitor significantly reduced levels of H3K9la and MyHC expression in myoblasts with lactate treatment (Figure 3C–F). Then we evaluated the levels of histone acetylation in the presence of lactate, also include the effects of the p300 inhibitor on histone acetylation with lactate treatment. And we found that lactate treatment significantly increased histone lactylation level rather than the histone acetylation level (Figure 1D and Figure S3a,b), indicating that lactate‐induced muscle differentiation might be mainly directed by histone lactylation, and the role of acetylation could be ruled out. These results might indicate a possible involvement of H3K9la in lactate‐induced myoblast differentiation.

### Identification of potential transcriptional targets of H3K9la

**Figure 4:**
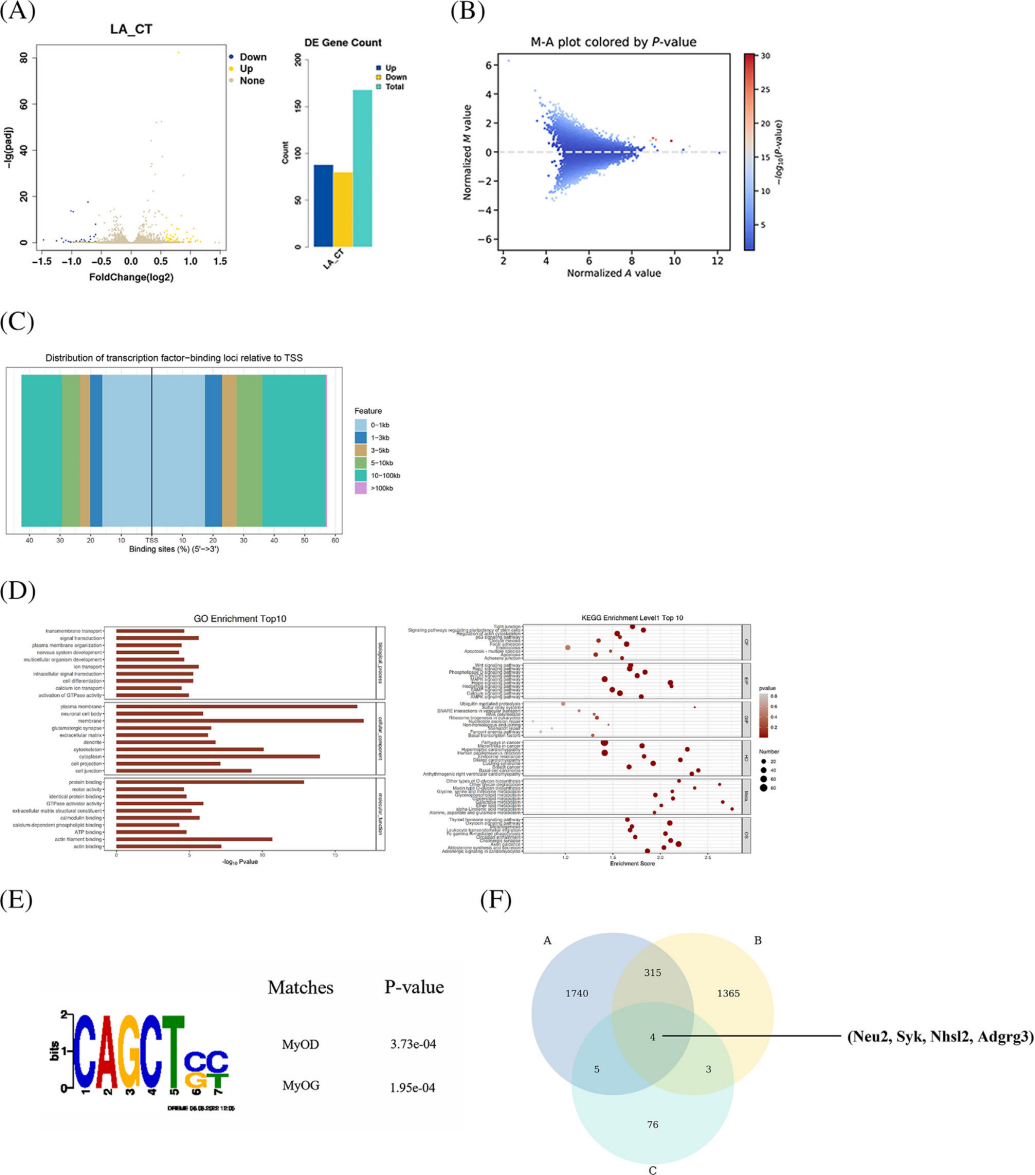
Identification of potential target genes by RNA‐seq and CUT&Tag associations analysis. (A) Volcano plot of differential genes between control and treatment groups in RNA‐seq analysis. (|Foldchange| > 1.5, *P*_adj_ < 0.05, are significantly differentially expressed genes.) (B) Volcano plot of CUT&Tag different peaks analysis. L‐1 VS CT‐1 (|M| > 0.585, *P* < 0.05 are significantly differentially expressed peaks.) (C) TSS analysis of H3K9la. (D) Genes significantly differentially enriched for bound peaks with H3K9la were used for GO analysis and genes significantly differentially enriched for bound peaks with H3K9la were used for KEGG analysis. (E) Motif analysis of lactate treatment group and compared with Tomtom database. (F) Venn diagram intersection of RNAseq up‐regulated genes and H3K9la enriched up‐regulated peaks annotated to promoter regions (L‐1 VS CT‐1, L‐2 VS CT‐2). Then we identified four genes (Neu2, Syk, Nhsl2 and Adgrg3) might be targets of H3K9la.

Next, we examined the levels of H3K9la modification during myoblasts differentiation at day 1, day 2 or day 3. Compared with cells without lactate treatment, the levels of endogenous H3K9la and global histone lactylation were significant higher in those treated with lactate in each day (Figure S2). To identify the target genes of H3K9la, we performed combined omics analysis using Cut&Tag and RNA‐seq. Based on the results of Fig S2c, the myoblasts cultured for 3 days were collected for Cut&Tag and RNA‐seq. The transcriptome analysis showed 168 significantly differential expressed gene sets, of which 88 genes were significantly up‐regulated and 80 genes were significantly down‐regulated between control group (CT) and lactate‐treated group (LA) (Figure S4 and Figure 4A).

We next performed differential analysis of annotated DNA peaks enriched with the H3K9la antibody by MAnorm software, and the results were displayed as a volcano plot (Figure 4B). Then, we performed transcription start site (TSS) analysis on the peaks and found that H3K9la‐enriched regions were mainly distributed around −1 to 1 kbp up and downstream of the TSS (Figure 4C), which is known as a typical binding site of modified histone. Also, we use chipeseeker software to analyse the distribution of annotated peaks on functional elements of genes. The result revealed that H3K9la was mainly enriched in the promoter region (Figure S5e), indicating a possible role of H3K9la in regulating genes transcription. Furthermore, we performed gene ontology (GO) analysis and Kyoto encyclopedia of genes and genomes (KEGG) analysis for genes with differentially enriched peak annotations in promoter regions, and the top 10 significantly enriched pathways were listed for each item; we found that some extracted GO terms and KEGG pathways are closely related to muscle differentiation associated processes, such as Hippo signalling pathway,^29^ regulating pluripotency of stem cells pathways, mTOR signalling pathways,^30^ cell differentiation and so on (Figure 4D). Also, we used Dreme software to predict the potential motif sequences, which was then compared with the known motif database in Tomtom website (https://meme‐suite.org/meme/tools/tomtom). Interestingly, we identified a conserved motif recognized by the myogenic regulatory factors (Figure 4E), including myogenic differentiation 1 (MyoD) and myogenin (MyoG). Using Venn diagram, we established the intersection of lactate up‐regulated differential expressed gene sets and the genes annotated to promoter regions, and four genes (Neu2, Syk, Nhsl2 and Adgrg3) were identified as potential targets of H3K9la (Figure 4F). Of the four genes, only Neu2 is related to muscle differentiation based on GO annotation and literature reports^15^, ^16^, ^17^, ^18^, ^19^, ^31^, ^32^; we thus tested whether Neu2 is involved in H3K9la‐mediated myogenesis.

### The binding of H3K9la in transcription start site activates Neu2 transcription in lactate‐treated myoblasts

**Figure 5:**
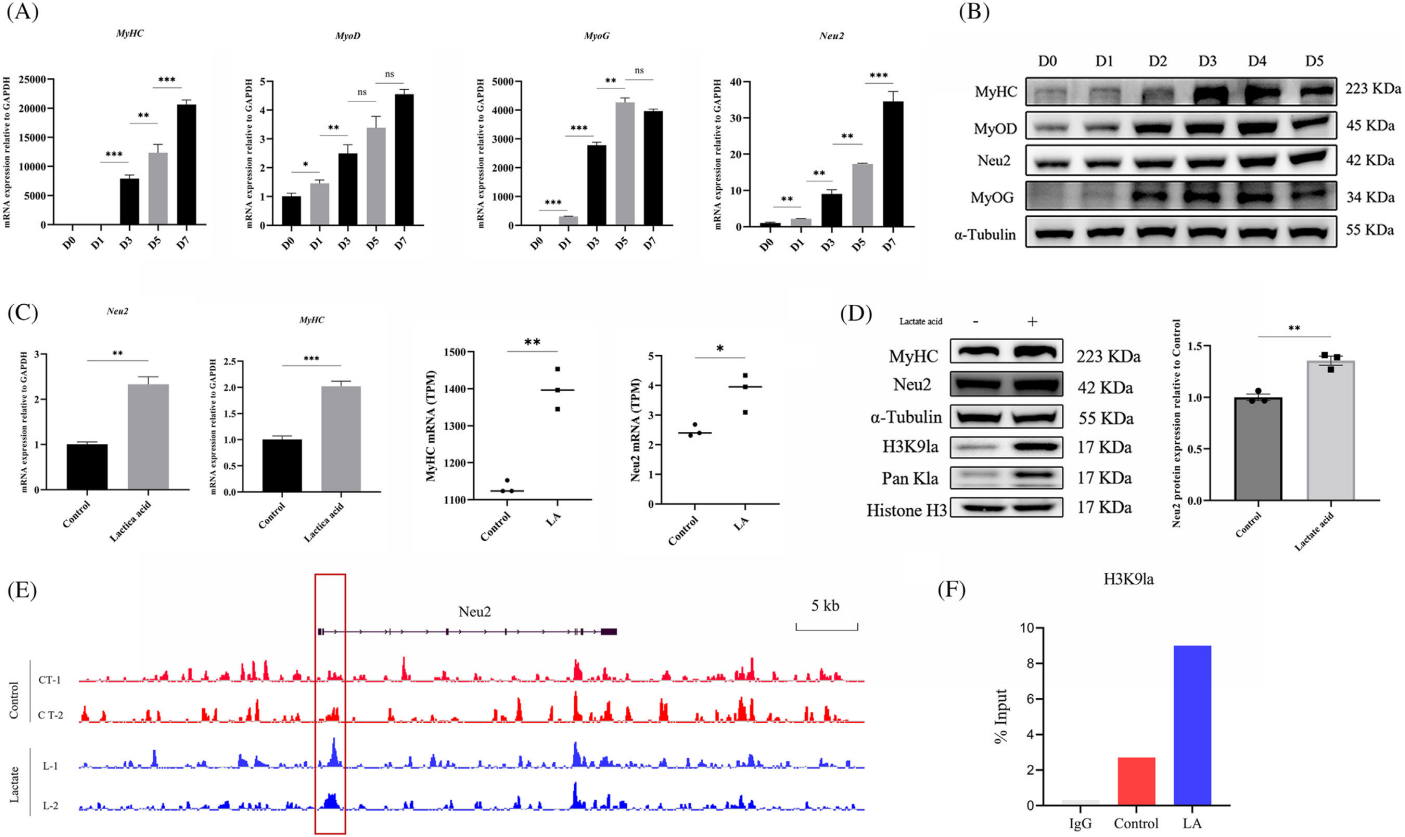
H3K9la activates Neu2 expression to promote myoblast differentiation. (A) qRT‐PCR analysis of mRNA expression of MyoG, MyHC, MyoD and Neu2 in differentiated C2C12 cells (D0, D1, D3, D5 and D7). Mouse β‐actin was used to normalize the gene expression levels as an endogenous reference gene during C2C12 differentiation. (B) C2C12 cells are differentiated for D0, D1, D2, D3, D4 and D5, then detected myogenesis protein level and Neu2 protein level by western blot. (C) qRT‐PCR analysis of mRNA expression of Neu2, MyHC on C2C12 cells at the day 3 of differentiation. *Neu2*, *MyHC* mRNA expression in C2C12 cells with lactate treatment or not (from RNA‐seq results). (D) C2C12 cells cultured with 15 mM lactate and induce differentiation for 3 days, then detected Neu2 protein level by western blot. And quantitation of the Neu2 protein expression level relative to α‐tubulin. (E) IGV views of H3K9la CUT&tag sequence data on Neu2. (F) ChIP–qPCR analysis of H3K9la binding to Neu2 promoter region; data represent three technical replicates from pooled samples. All data are expressed as mean values ± SEM; *n* = 3 biological replicates in each group. *P < 0.05, **P < 0.01, ***P < 0.001.

To explore the role of Neu2 in myoblast differentiation, we first examined changes of Neu2 transcript level and protein level at different stages of myogenesis. Our results are consistent with previous reports that Neu2 expression levels gradually increase during myoblast differentiation (Figure 5A,B). qRT‐PCR data showed that mRNA expression of Neu2 and MyHC was significantly elevated after 3 days of lactate (15 mM) treatment, consistent with the results obtained from the RNA‐seq analysis (Figure 5C). In addition, the expression of Neu2 protein was markedly increased after lactate treatment (Figure 5D). Based on the CUT&Tag sequencing results, we then used IGV software to delineate the binding signals of H3K9la in the *Neu2* gene to display that enrichment of H3K9la is elevated in the promoter region nearby TSS after lactate treatment (Figure 5E). Thus, we designed amplification primers for this region, and the ChIP‐qPCR results demonstrated that lactate treatment markedly increased binding of H3K9la in the TSS of *Neu2* (Figure 5F). Together, these results indicated that lactate‐derived H3K9la could promote *Neu2* transcription during myoblast differentiation.

### Inhibition of Neu2 activity impairs lactate‐induced myoblast differentiation

**Figure 6:**
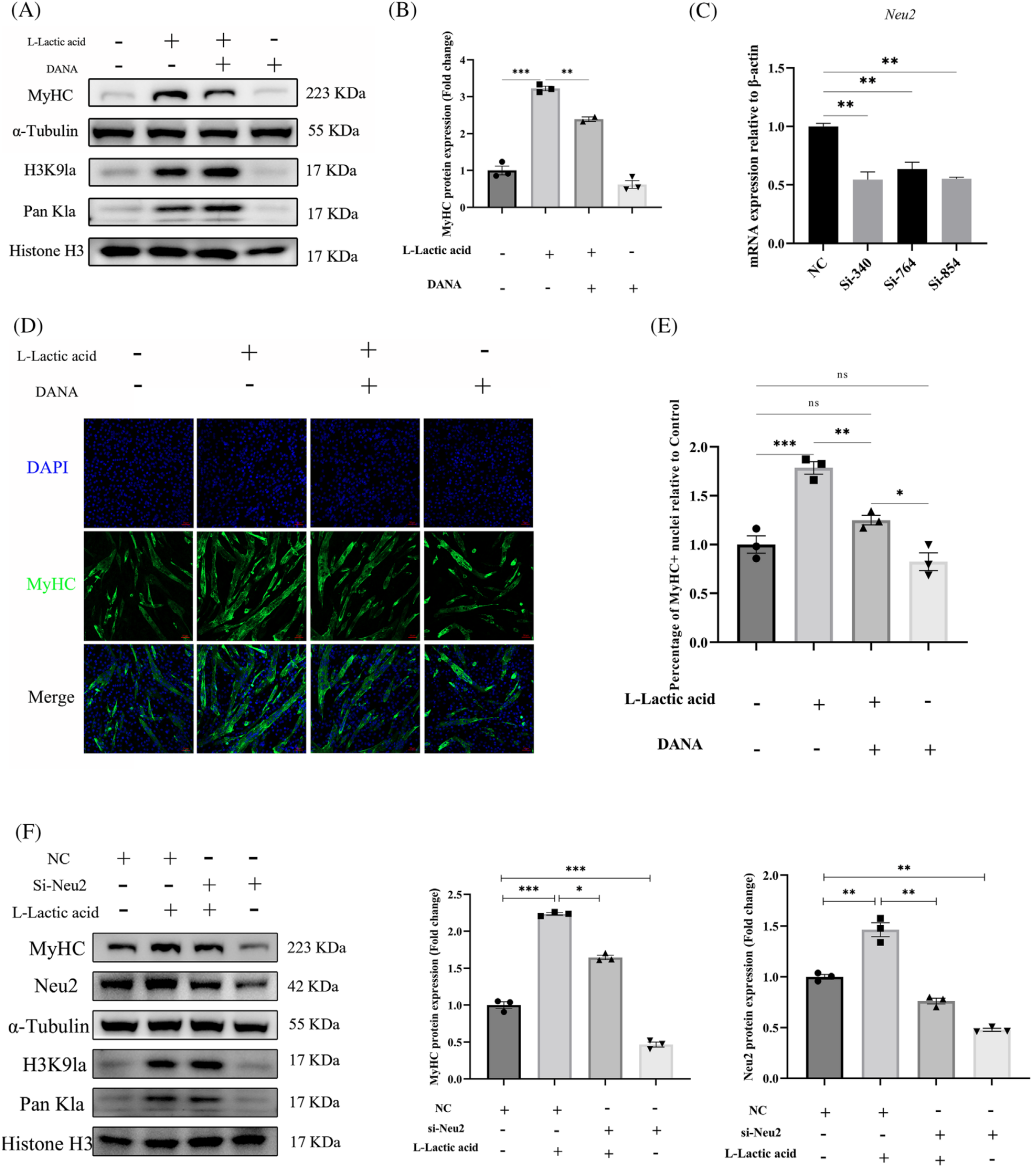
Inhibition of Neu2 expression impaired lactate‐derived myoblast differentiation. (A) After inhibit Neu2 function by DANA (50 μM), then cultured with 15 mM lactate and differentiation for 3 days. Then detected MyHC protein level by western blot. (B) Quantitation of the MyHC protein expression level relative to α‐tubulin in panel (A). (C) Verification of interference efficiency on Neu2 by RT‐PCR. Mouse β‐actin was used to normalize the gene expression levels as an endogenous reference gene during C2C12 differentiation. (D) Representative photographs of MyHC, immunofluorescence staining in C2C12 cells differentiated for 3 days under L‐lactate (10 mM) and DANA (50 μM) treatment. Scale bar = 100 μm. (E) Quantitation of the MyHC positive cells. (MyHC+ is the proportion of MyHC positive cells with at least one nucleus). (F) After knock down Neu2 expression by SiRNAs, then cultured with 15 mM lactate and differentiation for 3 days. Then detected MyHC, Neu2 and lactylation protein level by western blot. And quantitation of the MyHC, Neu2 protein expression level relative to α‐tubulin and histone H3. All data are expressed as mean values ± SEM; *n* = 3 biological replicates in each group. *P < 0.05, **P < 0.01, ***P < 0.001.

To determine whether Neu2 is required for lactate‐mediated myoblast differentiation, we treated cells with DANA, a specific inhibitor of Neu2. As shown in Figure 6A,B, DANA diminished MyHC protein expression despite lactate treatment. Consistent with this, the growing number of myotube induced by lactate treatment was decreased in the presence of DANA (Figure 6D,E). Moreover, we designed three pairs of siRNAs to silence Neu2 expression (Figure 6C). To improve the knockdown efficiency, we transfected myoblast with the mixture of these siRNAs. The differentiation level of myoblast up‐regulated by lactate treatment was significantly hampered following the inhibition of Neu2 expression (Figure 6F).

### Lactate injection facilitates muscle regeneration and histone lysine lactylation

**Figure 7:**
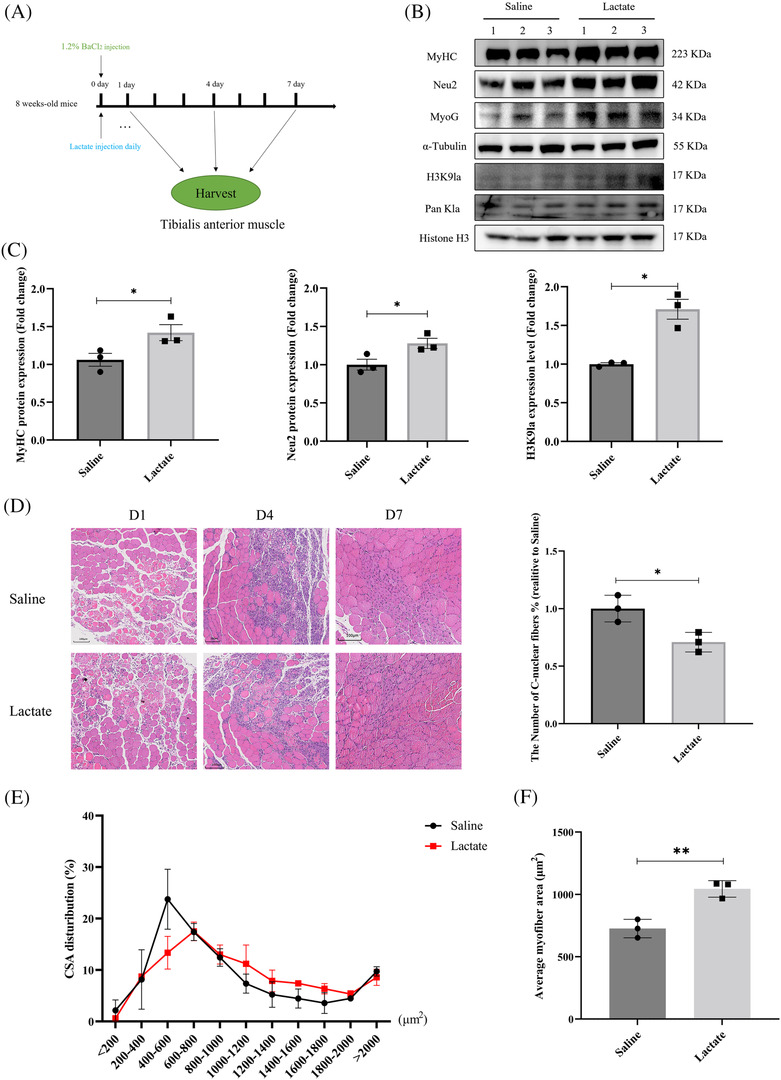
Lactate advance muscle regeneration with elevated histone lactylation. (A) Schematic diagram of lactate injection and muscle injury experiments. (B) TA muscle harvest on D7, then detected MyHC protein and histone lactylation level by western blot. (C) Quantitation of the MyHC, Neu2, H3K9la protein expression level relative to α‐tubulin and histone H3 in panel (B). Data are expressed as mean values ± SEM; *n* = 3 replicates in each group (mice = 6 in each group). *P < 0.05, **P < 0.01, ***P < 0.001. (D) Comparison of H&E staining for the regeneration of TA muscle following injury. Scar bar = 100 μm, 10×. And quantitation of the number of centre nuclear myofibres relative to saline at D7 (*P < 0.05, **P < 0.01; *n* = 3, 2‐monts‐old male mice, three sections per sample). (E) Quantitation of muscle fibre cross‐sectional area (CSA) distribution at D7. (F) Average of myofibres area. TA muscles after treatment with lactate and saline, 7 days after injury (*P < 0.05, **P < 0.01 *n* = 3, 2‐month‐old male mice, three sections per sample).

Regeneration of injured skeletal muscles is associated with myoblast differentiation. We thus employed a previously established model of skeletal muscles injury^33^ to further verify the relationship among lactate, histone lactylation and muscle differentiation in vivo. Briefly, tibialis anterior (TA) muscle of mice was intramuscularly injected with 1.2% BaCl_2_ to induce muscle damage (Figure 7A). For muscle regeneration, mice were subjected to intraperitoneal injection with sodium lactate once daily for 7 days (Figure 7A). TA muscle was collected at the indicated time points for western blot assay and H&E staining. As shown in Figure 7C, mice received lactate injection revealed significant higher levels of MyHC, MyoG, histone lactylation, H3K9la and Neu2 in TA muscle compared with that in the saline group (Figure 7B,C). The decrease of centronuclear myofibres went along with a decrease of smaller fibres and an increase of larger fibres, all indicative of improved skeletal muscle regeneration (Figure 7D–F).

## Discussion

Myoblast differentiation is coupled with metabolic reprogramming,^11^ as represented by a switch of glycometabolism from anaerobic glycolysis to oxidative phosphorylation, leading to alterations in the lactate level. However, the crosstalk between metabolic reprogramming and myogenesis remains to be explored. Accumulating evidence suggested that histone acylation might serve as a bridge connecting cell metabolism and differentiation. Histone lactylation is a novel form of acylation modification identified in recent years,^14^ which has been reported to mediate tumorigenesis and inflammation via regulating gene transcription.^14^, ^28^ However, little is known regarding whether lactate modification is involved in muscle differentiation. As reported, lactate could promote myoblast differentiation in vitro and muscle regeneration in vivo,^6^ although the underlying mechanism remains elusive. Interestingly, histone lactylation is sensitive to lactate concentration.^28^, ^34^ It prompted us to determine whether histone lactylation is involved in lactate‐induced myogenesis. Here, we found that lactate promoted myoblast differentiation with elevated histone lactylation, whereas inhibition of histone lactylation could block muscle differentiation. Taken together, our findings provide first evidence demonstrating the role of histone lactylation in regulating myogenesis. In addition, our data extend the mechanistic understanding of lactate‐induced myoblast differentiation.

Myogenesis is a tightly regulated process orchestrated by a set of epigenetic regulators, such as DNA methylation, histone modification, N6‐methyladenosine (m6A), and non‐coding RNA.^3^ In particular, histone modifications play a central role in muscle formation.^10^ For instance, histone H3K9 acetylation was recognized as a differentiation switch of myoblast. When myoblasts are induced to differentiate into myotubes, the promoters of muscle specific genes were deposited with the H3K9 acetylation mark.^13^ In this study, we revealed that the H3K9 lactylation‐mediated regulation of gene transcription is also responsible for myoblast differentiation. Our findings provide novel insights into the epigenetic mechanisms of muscle development.

Actually, lactylation occurs in both histone proteins and non‐histone proteins,^27^, ^35^ but it remains to be clarified if they function independently or synergistically. In this study, we observed that lactate‐induced lactylation modifications were mainly distributed in the nuclei, suggesting that histone lactylation rather than non‐histone lactylation is more likely to participate in myoblast differentiation. Several lactylation sites on histones, such as H3K4, H3K9, H3K18, and H3K27 have been recognized in human and mouse cells.^23^ Using lactylation site‐specific antibodies, we identified H3K9la as a candidate modification site required for muscle differentiation. However, we could not rule out the possibility that lactylation of non‐histone proteins in the nucleus might be involved. As such, we observed that lactate‐induced lactylation also occurred in the proteins beyond the molecular weight of histone (15–25 KDa). The possible role of non‐histone lactylation in regulating skeletal muscle differentiation has yet to be realized and requires further research.

Neuraminidase 2 (Neu2) is a cytosolic sialidase that have been reported to play an important role in skeletal muscle differentiation.^17^, ^18^, ^19^, ^31^ However, it remains unclear whether Neu2 is involved in lactate‐induced muscle differentiation. In this study, we first demonstrated that lactate treatment specifically increased the expression of Neu2 during myoblast differentiation. This finding holds significance as Neu2 is a positive regulator of myogenic differentiation, suggesting a potential association between Neu2 and lactate‐induced myoblast differentiation. In addition, we unveiled an enrichment of H3K9la in the TSS of Neu2, potentially contributing to the enhanced transcription of Neu2 driven by lactate. Of note, the promoter region of Neu2 contains two E‐box sequences, which are known as conserved binding sites for myogenic regulatory factors like MyoD and MyoG.^17^, ^36^ MyoD, an early determinant of myogenic differentiation, along with MyoG, a terminal determinant of muscle differentiation responsible for myoblast fusion and MyHC expression,^37^ have been previously shown to interact with P300, a probable mediator of histone acylation modifications in the vicinity of myogenic genes.^38^, ^39^, ^40^ Importantly, we identified that the promoter motif recognized by H3K9la coincided with that recognized by MyoD. As P300 served as a writer for both acetylation and lactylation,^23^, ^28^ it is conceivable that MyoD might recruit P300 onto the promoter of Neu2, thus facilitating lactylation modifications of histones in this specific region, leading to the opening of chromatin structure and thereby activating Neu2 transcription. However, the validation of this hypothesis requires further investigation.

There are similarities between histone lactylation modification and histone acetylation modification in regulating gene transcription,^9^ although we exclude the possibility that lactate treatment might induce histone acetylation modification before. As mentioned above, histone acetylation is also involved in the regulation of muscle differentiation. As such, nothing is known regarding whether there is a crosstalk between acetylation and lactation in this process. The solution towards these questions by future investigations will improve our understanding of the regulatory mechanisms of muscle differentiation.

It is well documented that resistance training stimulates muscle growth, and this anaerobic exercise produced large amount of lactate. Typically, the maximum concentration of lactate could reach up to 20 mmol/L during strenuous exercise. But few studies identify the mechanism of lactate‐regulated muscle growth during resistance training. Here, in this study, we provided novel evidence showing that lactate injection promoted muscle regeneration and histone lactylation in mice skeletal muscle, which uncovered a possible mechanism of lactate to manipulate muscle growth during exercise. Nevertheless, we acknowledge a limitation of this study, as we solely utilized male mice for our experiments. To establish the broader relevance and potential gender‐specific effects of the lactate/H3K9la/Neu2 signalling pathway, further studies involving female mice are required.

**Figure 8:**
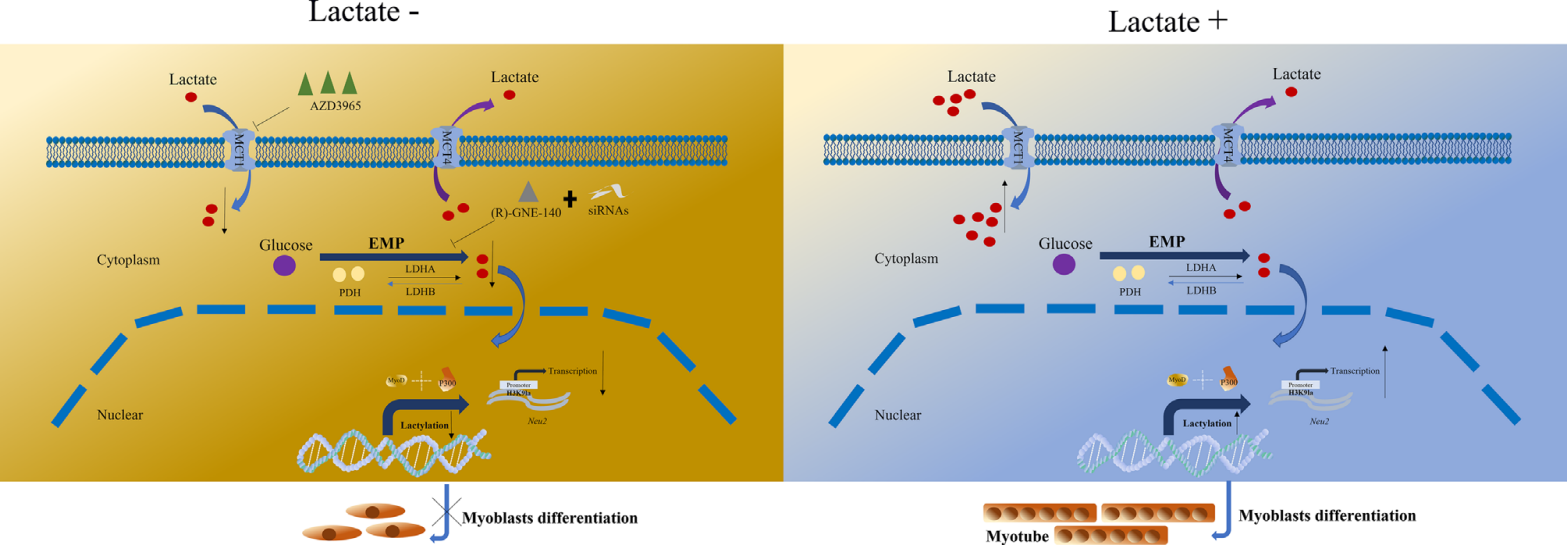
Schematic model of lactate‐derived histone lactylation promotes myogenesis. Lactate is taken up by myoblasts via MCT1 and then induced lactylation modification at the histone H3K9 site, which promotes Neu2 transcription and myogenesis.

In conclusion, this study revealed that lactate treatment promotes myoblast differentiation and muscle regeneration through H3K9la‐activated Neu2 expression (Figure 8). Our findings not only improve the epigenetic understanding of myogenesis but also provide potential therapeutic targets of muscle growth‐related diseases.

## Funding

This study was supported by the Jiangsu Agricultural Science and Technology Innovation Fund (CX (20) 2011), the Fundamental Research Funds for the Central Universities (KYT2023002 and KYQN2022030), The “JBGS” Project of Seed Industry Revitalization in Jiangsu Province (JBGS (2021) 026), National Natural Science Foundation of China (No. 31972571 and 31972564) and a Project Funded by the Jiangsu Province (BK20221512).

## Conflict of interest

The authors declare no conflicts of interest.

## Supporting information


**Figure S1.** Validation of interference efficiency of LDH‐siRNA.
**Figure S2.** Identify lactate derived histone lysine lactylation on myoblast.
**Figure S3.** Lactate treatment does not affect histone acetylation modification.
**Figure S4.** Quality estimates for RNA‐seq.
**Figure S5.** Quality estimates for CUT&TAG.

**Table S1.** The primers for qRT‐PCR.
**Table S2.** The primers for ChIP‐qPCR.
**Table S3.** Antibodies and their application.

## Data Availability

Raw data reported in this paper, including RNA‐seq, have been deposited in the Gene Expression Omnibus database under accession number GSE214250. CUT&Tag raw data have been deposited in the SRA database under accession number PRJNA885248.
