# Supplementary material for: Lactate promotes myogenesis via activating H3K9 lactylation‐dependent up‐regulation of Neu2 expression
Source: J Cachexia Sarcopenia Muscle. 2023 Nov 2;14(6):2851–65. doi: 10.1002/jcsm.13363 (PMC10751423; doi:10.1002/jcsm.13363)
Supplement: Supplementary file 1 — Figure S1. Validation of interference efficiency of LDH‐siRNA. Figure S2. Identify lactate derived histone lysine lactylation on myoblast. Figure S3. Lactate treatment does not affect histone acetylation modification. Figure S4. Quality estimates for RNA‐seq. Figure S5. Quality estimates for CUT&TAG. [file JCSM-14-2851-s001.docx]

**Supplementary figures**

**
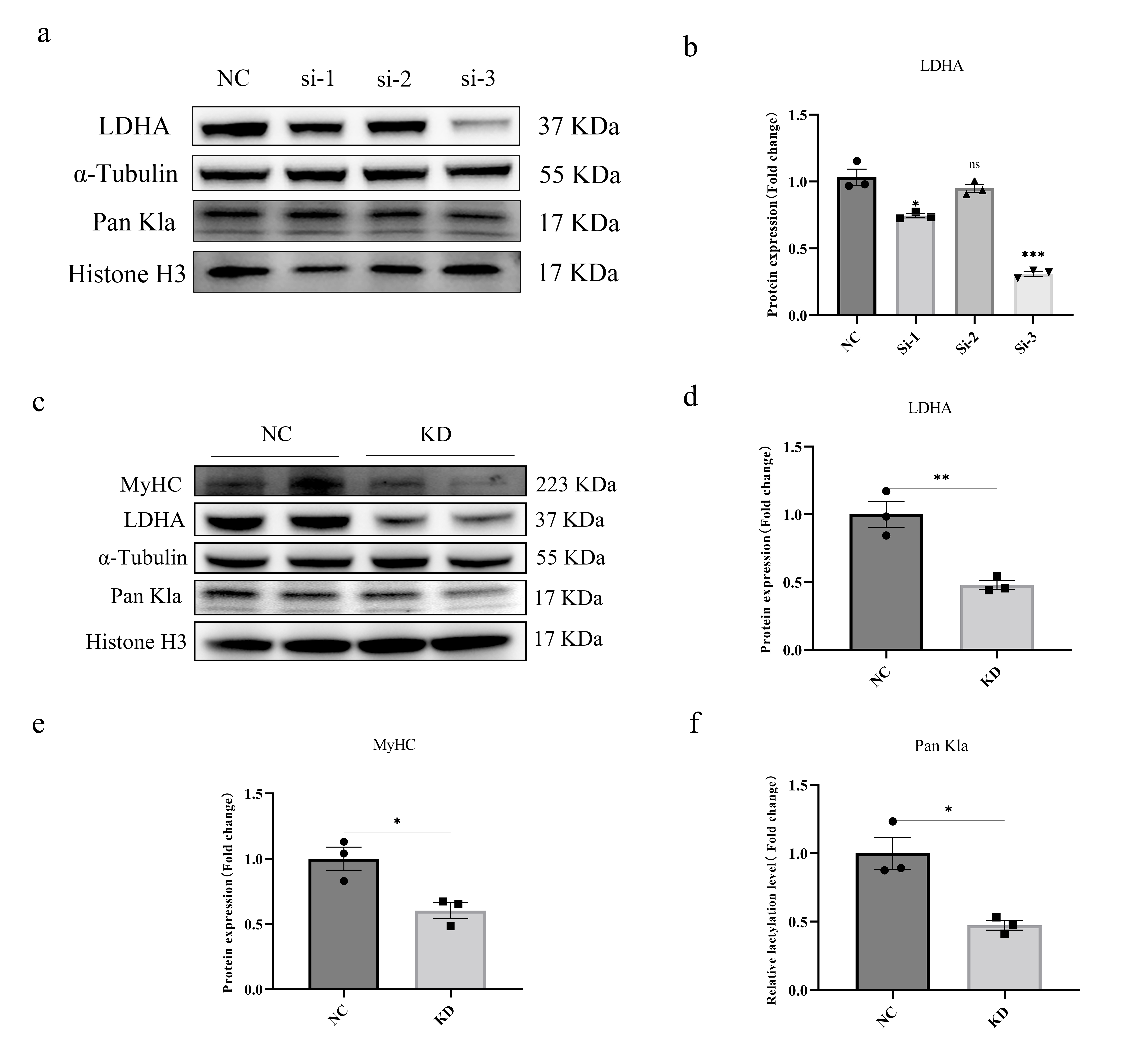
**

**Fig S1. Validation of interference efficiency of LDH-siRNA.**

**a.** Verification of interference efficiency on LDHA by Western blot. **b.** Quantitation of the LDHA protein expression level relative to α-Tubulin. **c.** Knock down LDHA expression by siRNA#3, then detected C2C12 differentiation level and histone lactylation by Western blot. **d-f.** Quantitation of the LDHA, MyHC, Kla protein expression level relative to α-Tubulin and Histone H3 in the c.

**
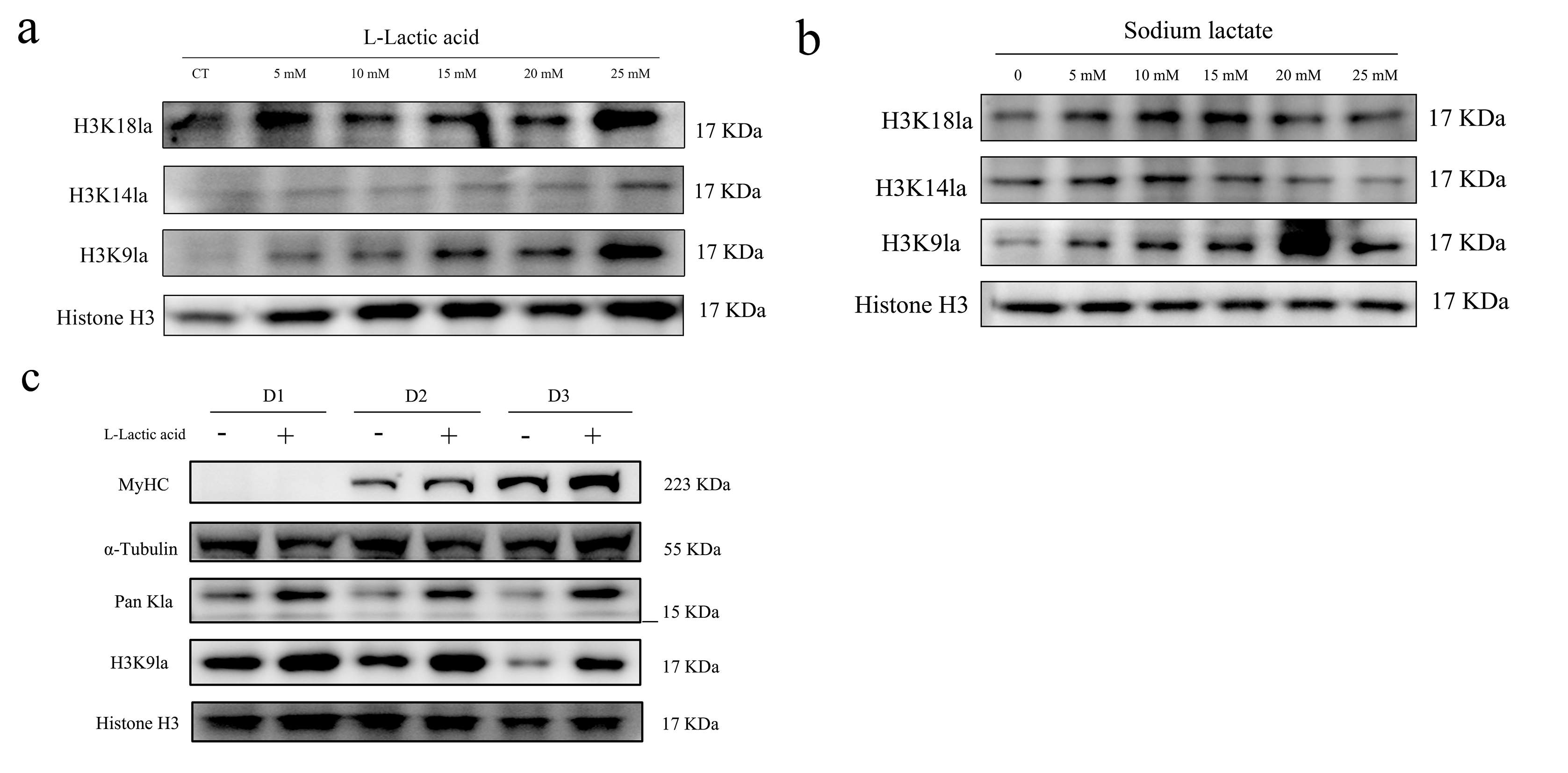
**

**Fig S2. Identify lactate derived histone lysine lactylation on myoblast.**

**a.** myoblast was cultured with different concentration of Lactic acid, and induce differentiation for three days, then detected histone lysine lactylation level by specific antibody (H3K9la, H3K14la, H3K18la) with Western blot. **b.** myoblast was cultured with different concentration of Sodium lactate, and induce differentiation for three days, then detected histone lysine lactylation level by specific antibody (H3K9la, H3K14la, H3K18la) with Western blot. **c.** C2C12 cells were treated with lactic acid (15 mM) and then induced to differentiate for 1, 2, and 3 days, respectively. Then detected MyHC, histone lactylation level by Western blot.


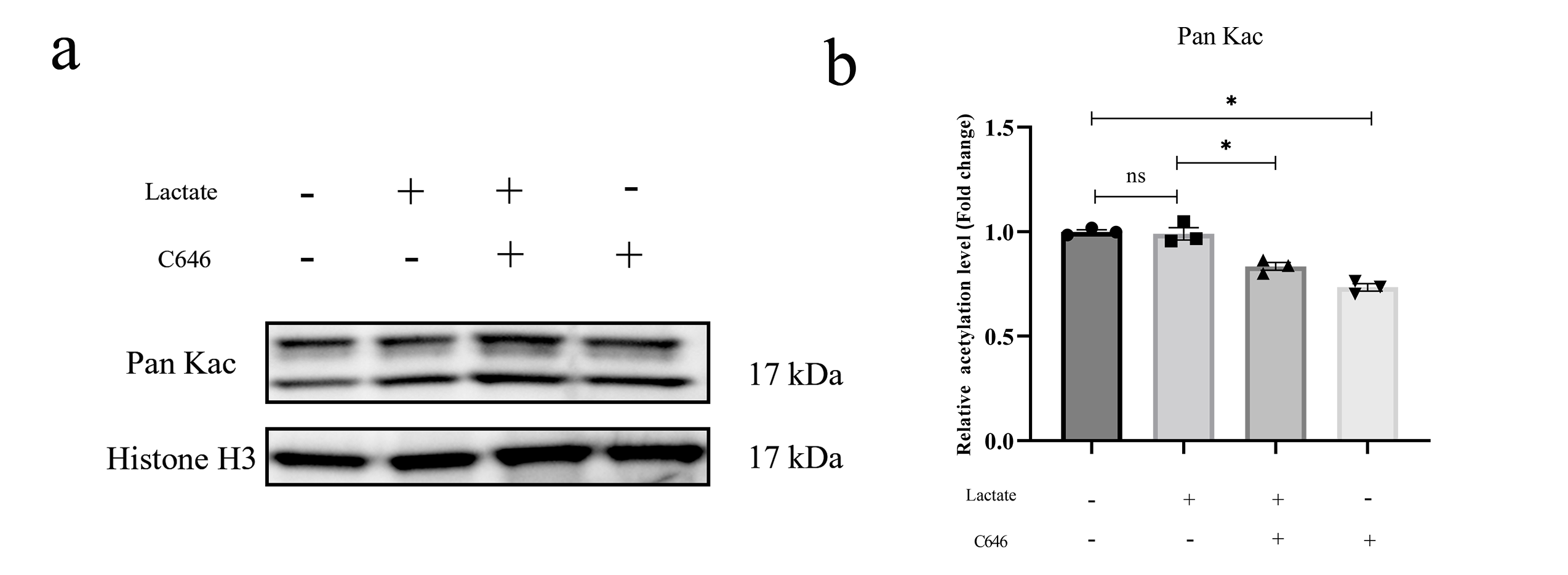


**Fig S3. Lactate treatment does not affect histone acetylation modification**

**a.** C2C12 cells treated with C646 (10 uM), then cultured with L-lactic acid (15 mM) for differentiation 3 days, then detected Histone acetylation by Western blot. **b.** Quantitation of the histone acetylation level relative to Histone H3 in the a. Data are expressed as mean values ± SEM; n=3 biological replicates in each group. *P < 0.05.


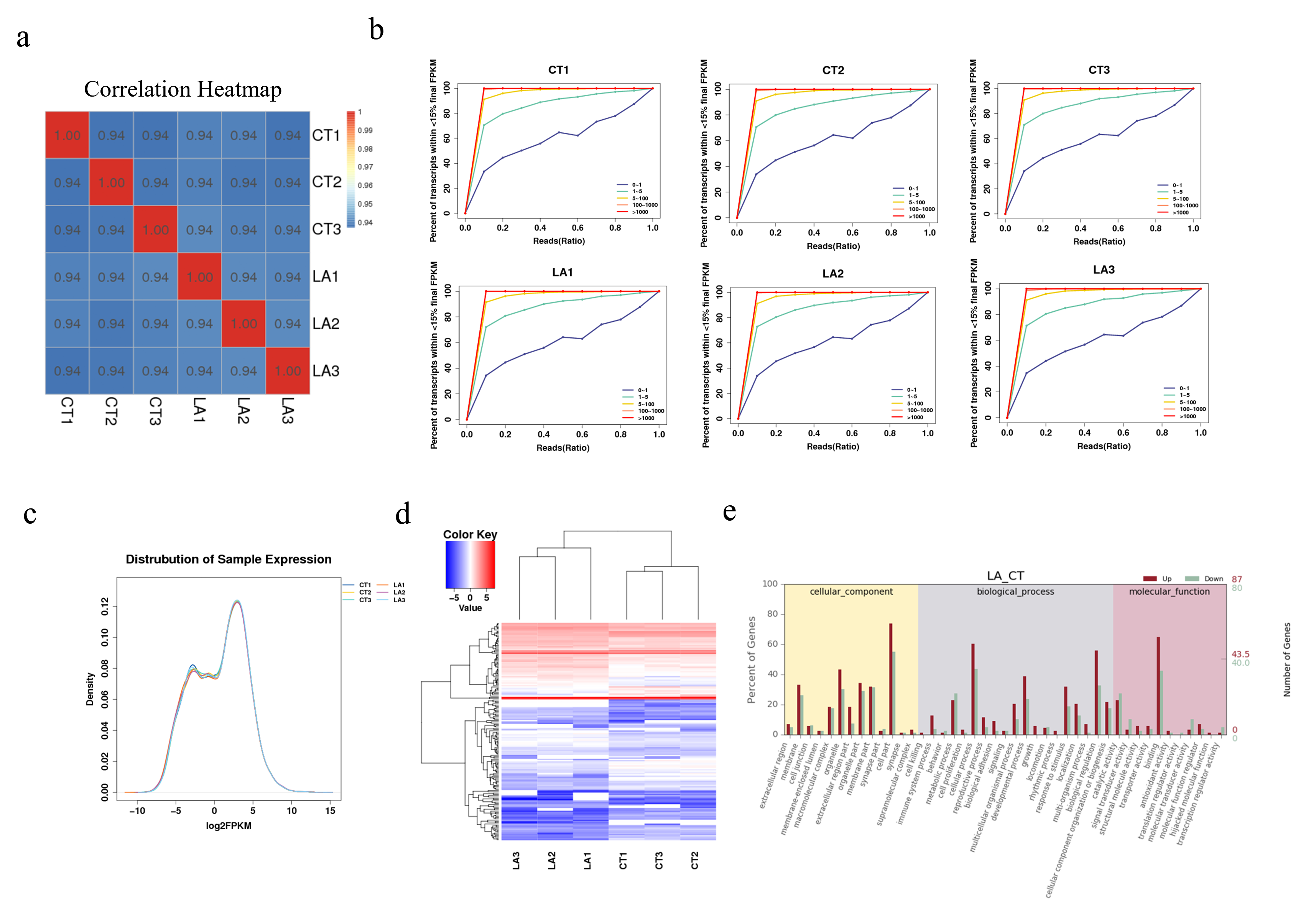


**Fig S4. Quality estimates for RNA-seq**

**a.** Correlation analysis between individual samples in RNA-seq analysis. **b.** Expression saturation analysis of individual samples **c.** Density of all sample’s genes expression. **d.** Heatmap of RNA-seq differential expression genes. **e.** Go analysis of differential genes in RNA-seq.

**
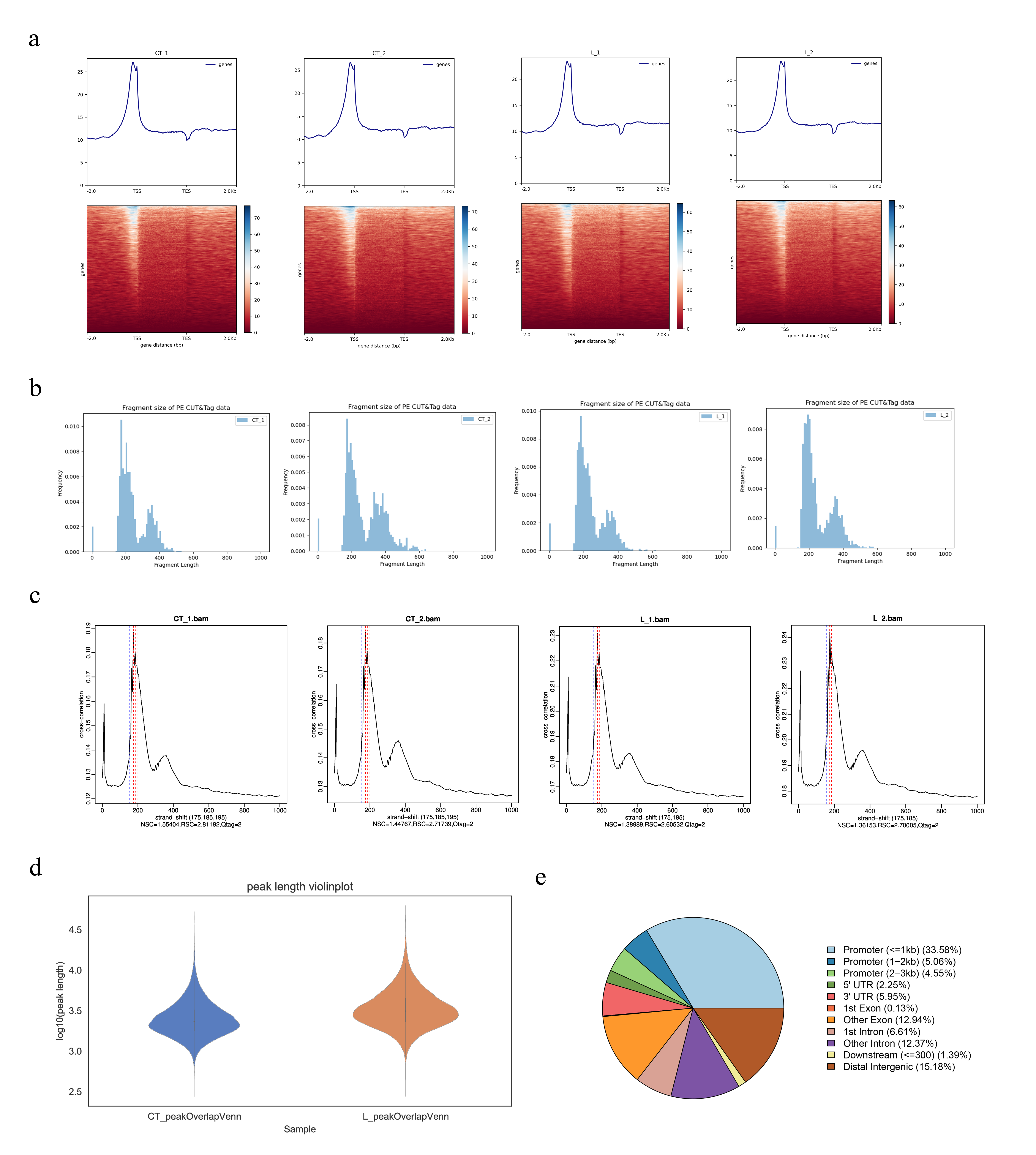
**

**Fig S5. Quality estimates for CUT&TAG**

**a.** The distribution map of CUT&TAG sequence reads on the genome. The horizontal axis is the TSS and the 2k region before and after the plot, and the vertical axis above is the mean of the reads abundance for the region. The bottom vertical axis represents a gene, the depth of color represents the relative number of reads aligned to the region, genes with similar distribution patterns are clustered together by clustering algorithms, thereby demonstrating the binding trend of a H3K9la across the entire gene, with a stronger binding trend being located indicating a greater ability of the transcription factor to bind at that region. **b.** The length of interrupted DNA fragment peak analysis. **c.** The analysis of H3K9la enrichment quality. **d.** H3K9la binds peaks length distribution diagram. **e.** Peaks distribution annotation on gene functional elements by chipseeker.
