# Supplementary material for: Lactate promotes myogenesis via activating H3K9 lactylation‐dependent up‐regulation of Neu2 expression
Source: J Cachexia Sarcopenia Muscle. 2023 Nov 2;14(6):2851–65. doi: 10.1002/jcsm.13363 (PMC10751423; doi:10.1002/jcsm.13363)
Supplement: Supplementary file 2 — Table S1. The primers for qRT‐PCR. Table S2. The primers for ChIP‐qPCR. Table S3. Antibodies and their application. [file JCSM-14-2851-s002.docx]

| Gene | Sequence | |
| --- | --- | --- |
| β-actin | | F: GCCTCACTGTCCACCTTCCA R: AGCCATGCCAATGTTGTCTCTT |
| MyHC | | F: CCAAAACCTACTGCTTTGTGGT R: GGGTGGGTTCATGGCATACA |
| MyOD | | F: CGAGCACTACAGTTGGCGACTAAGAT R: GCTCCACTATGCTGGACAGGCAGT |
| MyOG | | F: CCATCCAGTACATTGAGCGCCTACA R: ACGATGGACGTAAGGGAGTGCAGAT |
| Neu2 | | F: GGATGAGCACGCAGAGTTGAT R: GCCGTGTGACATTAACCTTAGT |

**Table S1 The primers for qRT-PCR**

**Table S2 The primers for ChIP-qPCR**

| Gene | Sequence | |
| --- | --- | --- |
| Neu2 Promoter | F: GCACATATGCCAAGCGGTTT | R: GCAGAAGGAAGCCGTCACT |

**Table S3 Antibodies and their application**

| Antibody name | Catalogue Number | Brand name | WB  (Dilution ratio) | IF  (Dilution ratio) | CHIP  (Dilution ratio) |
| --- | --- | --- | --- | --- | --- |
| Anti-rabbit IgG, HRP | ab6721 | Abcam | 1:2000 |  |  |
| Anti-mouse IgG, HRP | ab205719 | Abcam | 1:2000 |  |  |
| Rhodamine (TRITC)-conjugated goat anti-mouse IgG (H+L) | 33212ES60 | Yeasen |  | 1:200 |  |
| Alexa Fluor 488-Conjugated goat anti-mouse IgG(H+L) | 33206ES60 | Yeasen |  | 1:200 |  |
| Alexa Fluor 488-Conjugated goat anti-rabit IgG(H+L) | 33106ES60 | Yeasen |  | 1:200 |  |
| Anti-MyHC | MF-20 | DSHB | 1:500 |  |  |
| Anti-MyHC | SC-32732 | Santa cruz | 1:500 | 1:50 |  |
| Anti-Myogenin | SC-12732 | Santa cruz | 1:500 |  |  |
| Anti-MyOD | #13812 | Cell signaling | 1:1000 |  |  |
| Anti-Neur2 | YT7217 | Immunology | 1:1000 |  |  |
| Anti-pan kla | PTM-1401RM | PTM Bio | 1:1000 | 1:50 |  |
| Anti-H3K9la | PTM-1419RM | PTM Bio | 1:1000 |  | 1:50 |
| Anti-Histone H3 | #4499 | Cell signaling | 1:2000 |  |  |
| Anti-α-Tubulin | 2125S | Cell signaling | 1:2000 |  |  |
| Anti-pan Kac | 66289-1 | Proteintech | 1:2000 |  |  |
